# A Secure Medical Data Framework Integrating Blockchain and Edge Computing: An Attribute-Based Signcryption Approach

**DOI:** 10.3390/s25092859

**Published:** 2025-04-30

**Authors:** Tengyue Dou, Zhiming Zheng, Wangjie Qiu, Chunxia Ge

**Affiliations:** 1 The Second School of Clinical Medicine, Binzhou Medical University, Yantai 264003, China; 2 Health Blockchain Research Center, Binzhou Medical University, Yantai 264003, China; wangjieqiu@buaa.edu.cn; 3 Institute of Artificial Intelligence, Beijing Advanced Innovation Center for Future Blockchain and Privacy Computing, Beihang University, Beijing 100191, China; 4 Institute of Medical Artificial Intelligence, Binzhou Medical University, Yantai 264003, China; cxge01@bzmc.edu.cn

**Keywords:** attribute-based signcryption, blockchain, edge computing, access control, medical data security

## Abstract

With the rapid digitization of healthcare information, ensuring the security and privacy of patient data has become a critical research focus. This study introduces a novel Attribute-Based Signcryption (ABSC) framework combining blockchain and edge computing technologies to efficiently and securely manage medical data. The framework collects data via smart devices, which is then processed and encrypted at edge nodes and stored securely on the blockchain. Access to sensitive information is controlled with precision by predefined attribute sets, ensuring that only authorized users can retrieve the data. The experimental results demonstrate the significant advantages of this framework in improving data security, reducing computational overhead, and enhancing access efficiency.

## 1. Introduction

### 1.1. Background

In the digital age, healthcare information systems have undergone revolutionary changes, with patient health data increasingly stored and processed in electronic form. This transformation has not only enhanced the efficiency and quality of healthcare services but has also enabled precision medicine and research through data analytics. However, it has also introduced significant concerns regarding data security and privacy. The highly sensitive nature of patient data means that existing security measures, such as data encryption and access control, are insufficient to meet the complex demands of medical data processing [1,2]. This issue is particularly prominent in the Internet of Medical Things (IoMT) environment [3,4]. Therefore, ensuring the efficient and secure protection of data in the IoMT environment while safeguarding patient privacy has become an urgent challenge. As a result, improving data security to protect patient privacy has increasingly drawn the attention of researchers.

With the rapid development of healthcare informatization, the digital processing of medical data has become the norm. The widespread use of IoMT devices has significantly improved the efficiency of data collection, transmission, and storage. However, the sensitivity of medical data and its extensive distributed processing environment present significant security and privacy challenges. Although existing healthcare information systems have adopted traditional encryption methods and access control mechanisms, they often struggle to cope with the complexities introduced by emerging technologies like cloud computing and the Internet of Things (IoT) [5]. In particular, in environments with widespread data distribution, such as Electronic Health Record (EHR) systems, the sharing and transmission of patient data pose substantial privacy risks [2].

Traditional encryption methods play a critical role in medical data protection, but they also face many limitations. Walid et al. [3] compared various attribute-based encryption schemes, pointing out that traditional symmetric and asymmetric encryption methods perform poorly in handling complex data access control scenarios. Traditional encryption methods are often inadequate for supporting fine-grained access control based on user roles and attributes. To address this issue, Adeniyi et al. [6] proposed a blockchain-based smart healthcare system that focuses on data protection, which can significantly enhance privacy and security in medical data handling.

Attribute-Based Signcryption (ABSC) is a relatively new encryption technique that combines attribute-based encryption [7] with attribute-based signing mechanisms [8]. It defines data access permissions through attribute sets, enabling fine-grained access control [3,9]. By assigning permissions through attribute sets, ABSC allows users to access encrypted data based on their attributes, making it particularly suitable for protecting sensitive information such as medical data [10,11]. In healthcare applications, this scheme effectively handles user authentication and data encryption simultaneously, and its signing mechanism ensures data integrity. Sun et al. [12] developed a fine-grained attribute access control model for EHRs, enabling scalable and privacy-aware healthcare data exchange. However, while ABSC provides high security, its computational resource consumption is relatively high, which may make it unsuitable for low-power devices in medical environments [13]. Nevertheless, the efficient allocation and management of multiple encryption keys remains a challenge for this approach [14].

Although ABSC itself is a relatively new approach in encryption schemes, its application in healthcare, particularly for securing Electronic Health Records (EHR), has gained increasing attention. He et al. [4] mentioned that ABSC could ensure data privacy and security in cross-platform healthcare data sharing [15]. By introducing a revocation mechanism, user access permissions can be dynamically updated when user privileges change, further enhancing the security of data sharing [16,17]. Despite the excellent security provided by ABSC, its computational complexity remains a bottleneck, especially when it comes to large-scale medical data sharing [18,19].

### 1.2. Motivation and Contributions

The security and privacy of medical data have become critical issues in the era of digital healthcare, particularly with the rapid expansion of the Internet of Medical Things (IoMT). While blockchain and Attribute-Based Signcryption (ABSC) have been explored in several studies for securing healthcare data, the integration of these technologies within resource-constrained IoT environments, such as the IoMT, remains a challenging task. Patil et al. [20] implemented a chain-of-custody model with ABSC, ensuring secure traceability of medical data in multi-role scenarios.

This study proposes a novel framework that combines blockchain, edge computing, and ABSC in a unique way to address the specific needs of IoMT environments. While blockchain has been widely recognized for its ability to ensure data immutability and transparency, and ABSC has been proven to provide fine-grained access control, their integration into an efficient, decentralized system for the IoMT has not been fully explored [2,9]. Additionally, existing blockchain-based systems often rely on centralized cloud computing, which can introduce latency and security vulnerabilities.

Our contribution lies in the design of a hybrid framework that leverages both blockchain and edge computing to create a decentralized, secure, and scalable infrastructure for managing Electronic Health Records (EHR). The proposed system significantly reduces computational overhead and minimizes latency by performing encryption and decryption operations at edge nodes close to data sources, which is crucial for real-time medical data processing [4,21]. Moreover, the system incorporates ABSC to ensure that only authorized users, validated by specific attributes, can access sensitive medical data, making it highly suitable for multi-role scenarios within healthcare [12,20].

In comparison to previous works, this paper emphasizes the specific optimization of combining these technologies with edge computing to address IoMT’s unique challenges, such as resource constraints and the need for rapid, low-latency data processing. This work offers a comprehensive evaluation and comparison with existing solutions to highlight the advantages of our framework, including enhanced security, performance, and scalability. Furthermore, our detailed analysis demonstrates how edge computing improves the overall efficiency of blockchain-based EHR systems.

The contributions of this work are as follows:

- A novel framework that integrates blockchain, edge computing, and ABSC for securing and efficiently managing EHR data, designed specifically to meet the requirements of IoMT environments.
- A comprehensive evaluation that compares the proposed system with existing blockchain-based EHR solutions, showcasing its advantages in terms of security, scalability, and computational efficiency.
- A detailed analysis of the impact of edge computing on the performance of blockchain-based EHR systems, supported by real-world case studies and numerical simulations.

The structure of this paper is as follows: Section 2 introduces the related work used in this study. This section includes three sub-sections: Section 2.1 provides a general overview of the system design and theoretical foundations, Section 2.2 reviews relevant literature, and Section 2.3 outlines the core technologies and detailed system design. Section 3 describes the materials and methods, focusing on the system setup, core algorithms, and the overall workflow. Section 4 presents the discussion of the results of the study, including theoretical analysis and validation through numerical simulations. Section 5 provides a detailed discussion of the findings, focusing on the security model and threat model. It addresses key security aspects, including data confidentiality, integrity, access control, and potential threats, while also considering the system’s performance and efficiency. Finally, Section 6 concludes the paper, providing a summary of the research and suggesting future directions for continued work.

## 2. Related Work

### 2.1. Blockchain for Healthcare Data Security

Recent advancements in blockchain-based healthcare solutions have primarily focused on three critical dimensions:

1. Data integrity and auditability: Ghadi et al. [22] demonstrated a Hyperledger Fabric-based audit system achieving 99.7% tamper detection accuracy, though limited to 150 transactions per second (TPS). This work established baseline performance metrics for medical blockchain systems but overlooked real-time processing requirements. Hussien et al. [5] innovated with smart contract-driven dynamic policy updates, representing a 40% improvement over traditional RBAC systems. However, their centralized key management architecture (single Certificate Authority) introduced new vulnerabilities.
2. Decentralized access control: Mollah et al. [10] proposed a blockchain-edge hybrid model that reduced access control latency by 58% compared to pure cloud solutions. Their use of Ethereum smart contracts for policy enforcement demonstrated the feasibility of decentralized authorization. A breakthrough came from Quan et al. [13], whose 2023 framework achieved three times faster encryption throughput through edge-assisted computation offloading. Their multi-authority design mitigated single-point failures but increased signature verification overhead by 35%.
3. Cross-institutional sharing: Emerging solutions like Liu et al. [19] introduced a searchable ABE for EHR systems, enabling encrypted queries with 92% precision. However, their reliance on a trusted third party for key generation remained a limitation.

### 2.2. Evolution of ABSC Schemes

ABSC merges encryption and signing for attribute-based access. Attribute-Based Signcryption has undergone three generations of development:

1. First-generation schemes: Wang et al. [8] pioneered CP-ABSC using elliptic curves, achieving doctor/nurse role differentiation but with signature sizes growing linearly with attributes. Their 2024 work reduced verification time by 25% through optimized pairing operations. Hu et al. [9] established foundational security proofs for multicast communications, though their scheme required four pairing operations per decryption. Belguith et al. [23] proposed a Cooperative Attribute-Based Signcryption (C-ABSC) scheme that enables secure and collaborative communication between multiple IoT entities.
2. Lightweight innovations: Vijayakumar et al. [24] revolutionized the field with fog-assisted ABSC, cutting encryption latency from 380 ms to 120 ms (100 attributes) through computational task partitioning. Their 2024 PLOS One study demonstrated 98% reliability in ICU monitoring scenarios. Xiao et al. [25] achieved 40% bandwidth reduction via attribute-based forwarding, though at the cost of 15% higher CPU utilization on fog nodes. Sai Varshith et al. [17] leveraged kernel-level attribute enforcement to support dynamic policy updates in secure healthcare environments. Kibiwott et al. [26] addressed secure access control in large-scale IoMT systems through a verifiable and scalable ABSC architecture.
3. Dynamic attribute management: He et al. [4] introduced real-time revocation using timestamp attributes, reducing privilege escalation attacks by 60% but increasing storage overhead by 20%. Yang et al. [18] developed a mobile-optimized scheme with constant-size signatures, enabling secure sharing on smartphones with <100 ms latency. Xu et al. [14] proposed a secure data sharing scheme that integrates chaotic maps and blockchain, offering enhanced access control mechanisms similar to ABSC. Ma et al. [16] tackled dynamic access revocation via blockchain and ZK-Rollup, reducing user privilege escalation risks.

### 2.3. Real-Time Processing Frameworks

Edge computing reduces latency through proximal processing. The convergence of edge computing and medical security has produced three paradigm-shifting approaches:

1. Real-time processing frameworks: Xu et al. [21] achieved 80% faster emergency response times through fog-based pre-processing, though their multi-authority design increased key management complexity. Yang et al. [27] combined blockchain and ABSC to support real-time, consent-driven healthcare data sharing.
2. Hybrid encryption models: Yu et al. [28] introduced LH-ABSC for IoT devices, reducing signcryption energy consumption by 45% through lightweight cryptographic primitives. Hong et al. [29] demonstrated lightweight ABSC techniques that could enhance encryption performance in edge-assisted systems.
3. Federated learning integration: Liu et al. [30] made groundbreaking progress with BC-SABE, combining blockchain with searchable ABE to enable encrypted AI model training across hospitals while maintaining 92% diagnostic accuracy.

## 3. Materials and Methods

### 3.1. Preliminaries

This section introduces some fundamental technologies closely related to this research, including bilinear pairing, access control structures, and access tree structures. These technologies form the theoretical foundation for the subsequent system design and algorithm implementation.

#### 3.1.1. Bilinear Pairing

Let $G_{1},\;G_{2},$ and $G_{3}$ be three cyclic groups of prime order p. Let $g_{1}$ and $g_{2}$ be the generators of $G_{1}$ and $G_{2}$, and donate the bilinear mapping $e:G_{1}\times G_{2}\to G_{3}$. The bilinear mapping architecture proposed in this study satisfies the following properties:

1. Bilinearity: for all $g_{1}\in G_{1},\;g_{2}\in G_{2}\;\mathrm{and}\;a,\;b\in Z_{p}$, we have $e(g_{1}^{a},g_{2}^{b})={e(g_{1},g_{2})}^{ab}$, where $Z_{p}$ is the integer modulo prime p.
2. Efficient computation: for all $g_{1}\in G_{1}\;and\;g_{2}\in G_{2}$, there exists an efficient algorithm to compute $e(g_{1},g_{2})$.
3. Non-degeneracy: the generators $g_{1}$ and $g_{2}$ must satisfy $e(g_{1},g_{2})\neq 1$.

#### 3.1.2. Access Control Structure

Assume a set $P=\{P_{1},P_{2},...,P_{n}\}$ consisting of n data users, where a non-empty set $A\subseteq 2^{\left\{P_{1},P_{2},...,P_{n}\right\}}$ is a subset of this set. Let a monotonic set A be a non-empty subset of set P that contains the subset of all user groups that can access the data. Therefore, the monotonic non-empty set A is called an authorized set, while subsets not included in set A are referred to as unauthorized sets.

The core of the access control structure is the ‘monotonicity’. If a user group B has the right to access certain data (i.e., B∈A), then, as more users join this group, the new user group C(B⊆C) will still have access rights. In simple terms, if a qualified user group gains more members, its access rights will not decrease but only increase. In this case, the set is said to be monotonic.

#### 3.1.3. Access Tree Structure

Access tree structures play a key role in fine-grained access control, especially in CP-ABE, enabling complex access permission management.

The access tree structure typically consists of multiple nodes, each serving distinct roles and functions. These can be categorized into three types:

1. Root node: The root node is the top-level node in the access tree, representing the secret information to be protected (e.g., a key). Only users who meet specific conditions are allowed to access the data represented by this node.
2. Non-leaf nodes: These nodes are also called “threshold nodes” and function similarly to logic gates, such as “AND” or “OR”. Each non-leaf node has a threshold value. Permissions are granted only when the number of child nodes that meet the conditions reaches or exceeds the threshold.
3. Leaf nodes: These are the bottom-level nodes of the access tree. Each leaf node represents a specific attribute (e.g., “doctor”, “researcher”, or “age over 50”). Users must meet these attribute conditions to decrypt the corresponding data.

With these theoretical foundations in place, we then turn to the practical design and implementation of the system, where these concepts are applied to ensure secure and efficient data management. The next section will describe how the access control structure and the access tree are integrated into the system’s architecture to enforce strict data security policies.

### 3.2. The System

This section will provide a detailed description of the core algorithms and workflows of the system, with a focus on demonstrating how blockchain and edge computing technologies enable secure data storage and transmission. We will explain the interaction processes between the various modules and how the system ensures data integrity and privacy at different stages.

#### 3.2.1. System Settings

The system proposed in this study consists of six main components: smart devices, edge computing devices, service providers, trusted authorities, data owners, and data requesters. These modules work together to ensure the secure collection, transmission, storage, and access of data. The specific roles of these components and how they work together within the system are detailed below:

Smart device (SD): smart devices collect and preprocess data before ED-assisted encryption.

Edge device (ED): edge devices handle local storage while maintaining blockchain integrity.

Service provider (SP): service providers verify transactions and process authorized data requests.

Trusted authority (TA): trusted authority manages cryptographic identities offline after initialization.

Data owner (DO): DOs maintain data control through smart contracts.

Data requester (DR): DRs access data following predefined policies.

All are shown in the following table, Table 1.

**Figure 1:**
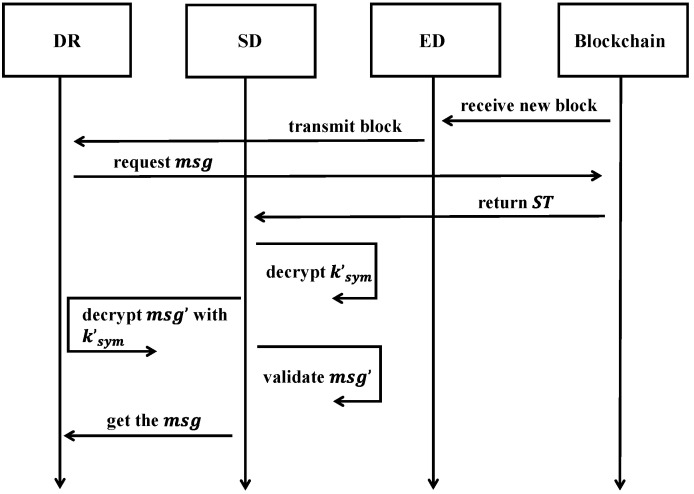
The roles and functions of the framework.

The details of the framework are as follows. First, the data owner (DO) collects their physiological data, such as heart rate and blood pressure, through a smart device (SD). After preprocessing, the SD encrypts the data using a lightweight encryption method. The encrypted data are then uploaded to a nearby edge device (ED) for storage. Next, the ED sends the encrypted data to the blockchain, where the encrypted message is recorded, ensuring the data are intact and tamper-proof. When a data requester (DR) wants to access the data, the DR queries the blockchain to find the corresponding encrypted message. Then, the DR sends a request to the DO through the SD, explaining why they need access to the data. The DO receives the request and decides whether to grant access based on the predefined access control policies. If the DO grants permission, the DR receives the decryption key and uses it to decrypt the encrypted data. During this process, the service provider (SP) helps process the data and uploads it to the blockchain, while the trusted authority (TA) is responsible for generating and distributing keys, ensuring user identities are verified and the data remains secure. This is shown in Figure 1.

#### 3.2.2. Algorithms

The system relies on several core algorithms to implement encryption, data access control, and secure communication. The primary algorithm used is SignCrypt, which combines signing and encryption operations to secure data. The algorithm is described in the following steps:

1. $Setup(1^{\lambda })\to (pk,mk)$: the setup algorithm takes a security parameter λ as input and produces public parameters pk along with a master key mk.
2. $KeyGen(pk,mk,S)\to (sk,k_{sign},k_{ver}$): with the public parameter pk, a master key mk, and a set of attributes S as inputs, this algorithm generates a secret key sk, a signing key $k_{sign}$, and a verification key $k_{ver}$ as outputs.
3. $SignCrypt(pk,M,T,k_{sign})\to ST$: given the public parameters pk, the plain-text M, the access tree T, and the signing key $k_{sign}$, the algorithm generates a signed ciphertext ST for the plain text according to the access tree T.
4. DeSignCrypt(ST,sk,S)→Mor⊥: using the signed ciphertext ST, the secret key sk, and the set of attributes S, the algorithm returns the plain text M if the attribute set S satisfies the access tree T. Otherwise, it returns the error symbol ⊥.

These algorithms work together to provide confidentiality, integrity, and access control, ensuring that only authorized users can access the encrypted medical data.

### 3.3. Scheme of the System

In this section, we will debate the specific construction process of this healthcare system based on the aforementioned algorithms. To ensure information security, the system design is divided into four key phases: system setup, entity registration, data collection and upload, and data query. The following sections will provide a detailed explanation of the implementation of each phase and its role in ensuring data security.

#### 3.3.1. System Setup

The trusted authorities (TAs) execute the Setup algorithm to derive the public parameters pk. Typically, the TA is involved only during the system’s initialization and entity registration phases. After these steps are completed, the TA operates offline. Initially, for a specific service provider with a pseudonymous identity id, the algorithm generates three cyclic groups, $G_{1}$, $G_{2}$ and $G_{3},$ of prime order p based on the security parameter λ. Then it generates the generators $g_{1}$ for $G_{1}$ and $g_{2}$ for $G_{2}$, followed by an efficient bilinear map $e:G_{1}\times G_{2}\to G_{3}$. Next, two random exponents $\alpha ,\beta \in Z_{p}$ are selected. The mk is derived from $mk=(\beta ,\;g_{2}^{\alpha })$. Then, the hash functions $H_{1}:{\left\{0,1\right\}}^{*}\to {\left\{0,1\right\}}^{\lambda }$ and $H_{2}:{\left\{0,1\right\}}^{*}\to Z_{p}$ are chosen. Following this, the TA computes $h=g_{1}^{\beta }$ and $t={e(g_{1},\;g_{2})}^{\alpha }$. Finally, the TA publishes the public parameters pk to all relevant entities on the blockchain network, which are structured as follows:

(1) $$pk=(p,G_{1},G_{2},H_{2},g_{1},g_{2},h,t)$$

#### 3.3.2. Entity Registration

When the service providers (SP), data owners (DO), or data requesters (DR) participate in the blockchain network, they must go through the registration phase, during which the TA verifies their identity. After successful verification, the TA runs the KeyGen algorithm. First, the TA selects a random value $r_{enc},\;r_{sign}\in Z_{p}$ and computes $D_{enc}=g_{2}^{\frac{\alpha +r_{enc}}{\beta }}$, $k_{sign}=g_{2}^{\frac{\alpha +r_{sign}}{\beta }}$ and $k_{ver}=g_{2}^{r_{sign}}$. Here, $k_{sign}$, $k_{ver},$ and sk are considered the signing key, verification key, and decryption key, respectively.

Additionally, the TA calculates $D_{j}=g_{2}^{r_{enc}}\cdot g_{2}^{H_{2}(j)∙r_{j}}$ and ${D^{\prime }}_{j}=g_{1}^{r_{j}}$, where $r_{j}\in Z_{p}$ is another randomly selected value for each attribute j∈S. Then, the TA returns the asymmetric secret key $sk=(D_{enc},\forall j\in S:D_{j},{D^{\prime }}_{j})$. Finally, the TA securely distributes the signing key $k_{sign}$ to the participating SPs through a secure communication channel, and the decryption key sk is assigned to the smart devices

(SD) with the attribute set S. SDs that are not connected to the TA can safely pre-store the key in the device.

**Figure 2:**
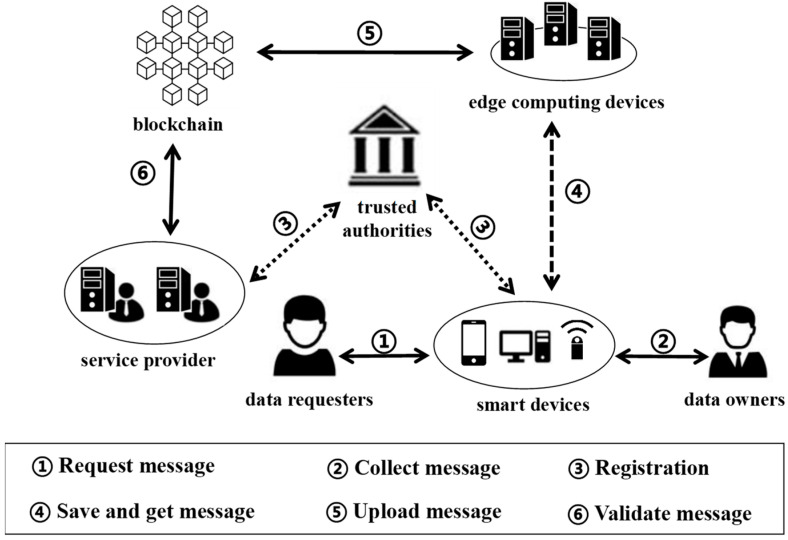
The framework of the registration.

The framework of the registration can be shown in Figure 2.

#### 3.3.3. Data Collection and Uploading

To send messages to the SDs’ blockchain network, the registered SP generates a random symmetric key $k_{sym}$ and uses $k_{sym}$ to encrypt the msg, resulting in a ciphertext $C_{msg}$. The SP then defines an access tree structure T, which represents a group of SDs that meet the access policy. Through T, the SP controls which SDs can access the encrypted message.

Each non-leaf node within the access tree T defines a threshold gate, which is governed by the number of child nodes and a corresponding threshold value. Leaf nodes correspond to attributes. For any internal node x in the T, let ${num}_{x}$ denote the number of child nodes and $k_{x}$ represent the threshold value, where $1\leq k_{x}\leq {num}_{x}$. For instance, if $k_{x}$ = 1, the gate performs an “OR” operation, opening if at least one child node meets the criteria. When $k_{x}$ = ${num}_{x}$, it operates as an “AND” operation, requiring all child nodes to satisfy the condition for it to activate.

In this study, we define the function index() to represent the order of the leaf nodes in the access tree, helping to assign unique values from 1 to num. We also define the set attr(x) as the attribute of the leaf node x. Once T is defined, the SP follows a detailed procedure to signcrypt $k_{sym}$ under the tree T.

First, after the SD collects the data from the DO, the SP runs the SignCrypt algorithm. This algorithm selects a polynomial $q_{x}$ for each node x, including the leaves in T. These polynomials are chosen from top to bottom, starting at the root node R. Starting from R, the algorithm randomly selects a value $s\in Z_{p}$ and assigns $q_{R}(0)=s$. Then, it randomly selects values $d_{x}$ from $Z_{p}$ to fully define $q_{x}$. For any other node x, it assigns $q_{x}(0)=q_{par.(x)}(index(x))$ and randomly selects values $d_{x}$ from $Z_{p}$ to fully define $q_{x}$. Let Y represent the set of leaf nodes in the access tree T. The SP randomly selects a value $\zeta \in Z_{p}$ and computes the following formulas: $\tilde{C}=k_{sym}⊕t^{s},\;C=h^{s},\forall y\in Y:C_{y}=g_{1}^{q_{y}(0)},{C^{\prime }}_{y}=g_{1}^{\left(H_{2}(attr(y))\cdot q_{y}(0)\right)},\;\delta ={e(C,\;g_{2})}^{\zeta },\;\pi =H_{1}(msg)+H_{2}(\delta ),\omega =g_{1}^{s},\;\psi =g_{2}^{\zeta }\cdot {\left(k_{sign}\right)}^{\pi }$.

Thus, the signcrypted ciphertext ST under the tree T is formed as

(2) $$ST=(T,\;\tilde{C},\;C,\;\forall y\in Y:\;C_{y},{C^{\prime }}_{y};\;\omega ,\;\pi ,\;\psi )$$

Finally, given the SP’s public key pk, pseudonymous identity id, the block hash $h_{2}$, and the signcrypted ciphertext ST (including the SP’s signature π), the record is stored and sent to the blockchain network for verification. The structure of the record is as follows.

(3) $$R=(pk,id,h_{2},ST)$$

**Figure 3:**
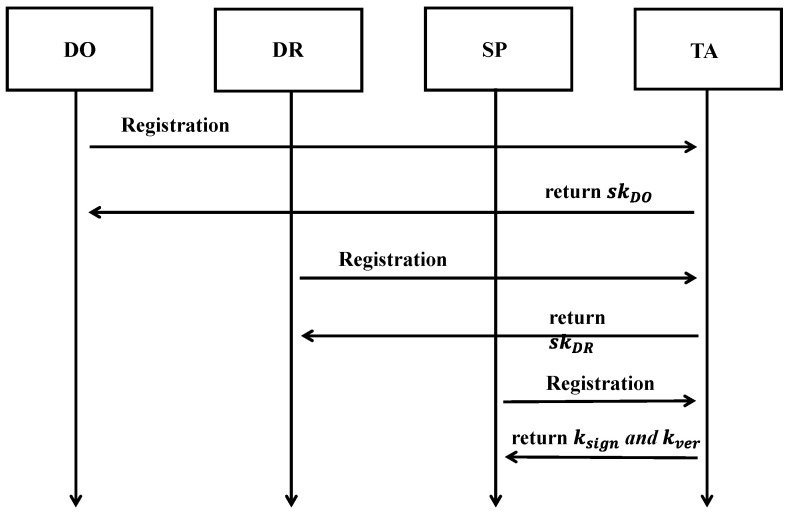
The framework of data collection and uploading.

The framework can be seen in Figure 3.

#### 3.3.4. Data Query

After receiving a new block, the ED can transmit the blockchain header to the SD, enabling the SD to decide whether a pull request should be initiated. If deemed necessary, the SD proceeds to retrieve the signed ciphertext ST and the signature π from the specific block. At this point, the data requester (DR) can initiate a data query for the required information by sending a request to the data owner (DO). Since access control is set up through the tree access structure, only DRs who meet the DO’s access structure conditions can successfully submit a request.

Once the SD obtains ST and π from the blockchain, it begins decrypting the data to extract the symmetric key $k_{sym}$, followed by retrieving the related message msg. The SD then validates the message integrity and proceeds with signature verification, as detailed below.

Initially, the SD executes the DeSignCryption algorithm. This recursive algorithm DecryptNode(ST, sk, x) accepts pk, ST, and the sk as inputs. The sk includes a set of attributes and a node x from the access tree T. If x is a leaf node, let i=attr(x). In this case, the algorithm returns the following result:

(4) $$DecryptNode(ST,\;sk,\;x)=\left\{\begin{array}{c} {e(g_{1},\;g_{2})}^{r_{enc}q_{x}(0)},i\in S\\ ⊥\;\;\;\;\;\;\;\;\;\;\;\;\;\;\;\;\;\;\;\;\;\;\;,otherwise\; \end{array}\right.$$

On the other hand, if x is a non-leaf node, the algorithm initially invokes the function DecryptNode(ST, sk, z) for each child node z of x, storing the results as $F_{z}$. Let $S_{x}$ represent the set of child nodes of x, with arbitrary size $k_{x}$, and include all child nodes z where $F_{z}\neq ⊥$. If such a set $S_{x}$ exits, the algorithm calculates $F_{z}$ as below:

(5) $$F_{x}=\prod_{z\in S_{x}}F_{z}^{{∆}_{i_{z},{S^{\prime }}_{x}}(0)}={e(g_{1},\;g_{2})}^{r_{enc}q_{x}(0)}.$$

where $i_{z}=index(z),{andS^{\prime }}_{x}=\{index(z)||z\in S_{x}\}$.

The DeSignCryption algorithm subsequently invokes the function on the root node r of the access tree. The SD can retrieve $k_{sym}$ provided it holds the correct attribute set S. If the conditions are met, the value is set as: $DecryptNode(ST,\;sk,\;r)=A={e(g_{1},\;g_{2})}^{r_{enc}q_{r}(0)}={e(g_{1},\;g_{2})}^{r_{enc}s}$. After decryption, the decrypted symmetric key ${k^{\prime }}_{sym}$ is obtained using the following calculation:

(6) $$\frac{\tilde{C}}{\frac{e(C,D_{enc})}{A}}=\frac{{{k^{\prime }}_{sym}\cdot \;e(g_{1},\;g_{2})}^{\alpha s}}{{e(g_{1},\;g_{2})}^{\alpha s}}={k^{\prime }}_{sym}.$$

Next, the SD also computes: $\delta^{\prime }=\frac{e(C,\;\psi )}{{\left(e(\omega ,{\;key}_{ver})∙\tilde{A}\right)}^{\pi }}$, where $\tilde{A}=\frac{e(C,{\;D}_{enc})}{A}$. Once ${k^{\prime }}_{sym}$ is retrieved, the SD uses it to decrypt the message $msg^{\prime }$. The SD then calculates $H_{1}(msg^{\prime })+H_{2}(\delta^{\prime })$. If the final result matches π, the SD confirms that the msg has not been tampered with.

**Figure 4:**
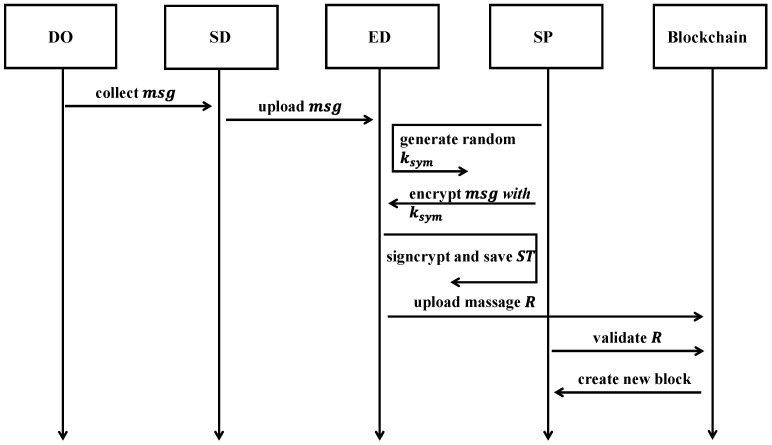
The framework of data query.

The framework is shown in Figure 4.

## 4. Results

### 4.1. Implementation Environment

To evaluate the performance of the proposed attribute-based signcryption (ABSC) scheme, we developed a system prototype using Python 3.6 and established a local network between the host, virtual machines, and Raspberry Pi (RPi) devices (Lenovo Group Limited, Beijing, China) using Oracle VirtualBox’s network bridging feature, enabling on-chain operations. The experimental platform included a host with an Intel i5 processor (Lenovo Group Limited, Beijing, China) and RPi devices equipped with 1 GB of RAM to simulate a resource-constrained Internet of Medical Things (IoMT) environment. The system’s performance was primarily evaluated by adjusting the number of attributes.

Before presenting the experimental results, we first detail the methods and experimental setup. In the experiment, we focused on several aspects, such as the choice of network architecture, the configuration of edge devices, and how blockchain and signcryption operations were deployed within this environment. For repeatability, we standardized the setup, using fixed hardware and software versions, and ensured network latency and resource consumption were kept within reasonable limits.

Subsequently, we adjusted the number of attributes to observe how the system’s performance changed. Specifically, we measured key indicators such as operation latency, computational load at edge nodes, and bandwidth consumption for data transmission across the network.

### 4.2. Theoretical Analysis

In this section, we compare the computational and communication overhead of our proposed scheme with existing schemes (references [3,24,31]) in four key stages: system initialization, registration, signcryption, and designcryption. We define $T_{e}$ as the time required for exponential operations, $T_{h}$ as the time for hash function operations, $T_{p}$ as the time for bilinear pairing operations, and $T_{m}$ as the time for multiplication operations. We define |N| as the total number of attributes within the attribute set.

As shown in Table 2, we compared the computational overhead at various stages of our scheme and the previous research scheme. The results show that our scheme achieves higher efficiency in several key stages. In the system initialization stage, our scheme has a fixed time of 3$T_{e}\;$ + $T_{p}$, which is more efficient than the schemes in references [3,24], where the initialization time depends on |N|. The registration phase in our scheme is (3 + |N|)$T_{e}$ + |N|$T_{h}$, which also scales more efficiently compared to the others. However, in the registration stage, our scheme optimizes efficiency by reducing the use of exponential operations and hash functions compared to previous research.

In the registration, signcryption, and designcryption stages, the computational overhead increases for both schemes as the number of attributes grows. However, when the number of attributes is large, our scheme demonstrates a slower growth in the overhead for exponential and multiplication operations, resulting in higher efficiency. Additionally, our solution offloads a significant portion of computations to edge devices, reducing the computational demands on local devices. This allows resource-constrained smart devices to process data more efficiently.

### 4.3. Numerical Simulations

We developed a system prototype using Python 3.6 and established a local network between the host, virtual machines, and RPi devices using Oracle Virtual Box’s network bridging feature, enabling on-chain operations. The experimental platform included a host with an Intel i5 processor and RPi devices equipped with 1 GB of RAM to simulate a resource-constrained IoMT environment. The system’s performance was primarily evaluated by adjusting the number of attributes.

**Figure 5:**
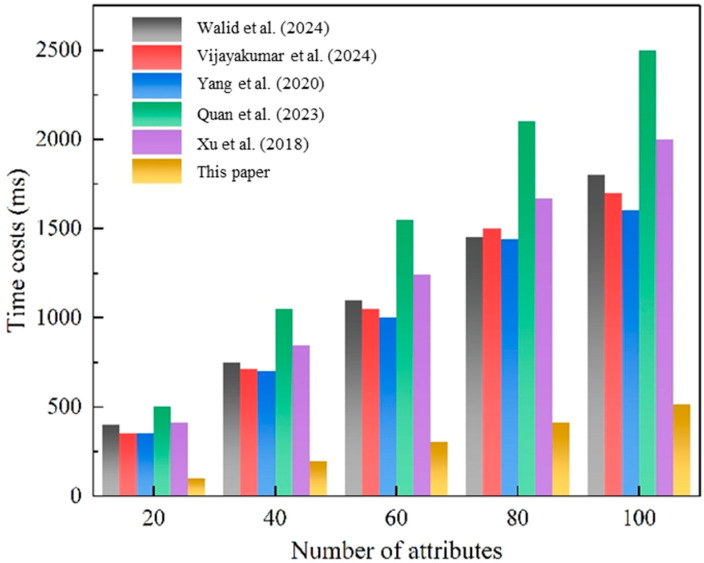
Signcryption on block creation and validation phase. This figure compares the time overhead of the signcryption process during the block creation and validation phase. The performance metrics are based on the framework proposed by Walid et al. [3], Vijayakumar et al. [24], Yang et al. [18], Quan et al. [13] and Xu et al. [21].

Figure 5 compares the signcryption time costs (in milliseconds) of different schemes as the number of attributes increases (from 20 to 100). While all schemes exhibit rising time costs, our proposed method shows a much slower growth rate. Notably, at 100 attributes, our solution completes encryption in 500 ms, representing a much better performance improvement than existing approaches (2000 ms for reference [3], 1800 ms for reference [24], and 1500 ms for reference [18]). This 67–75% reduction in computational overhead stems from our optimized ABSC protocol and intelligent workload distribution across edge computing nodes.

**Figure 6:**
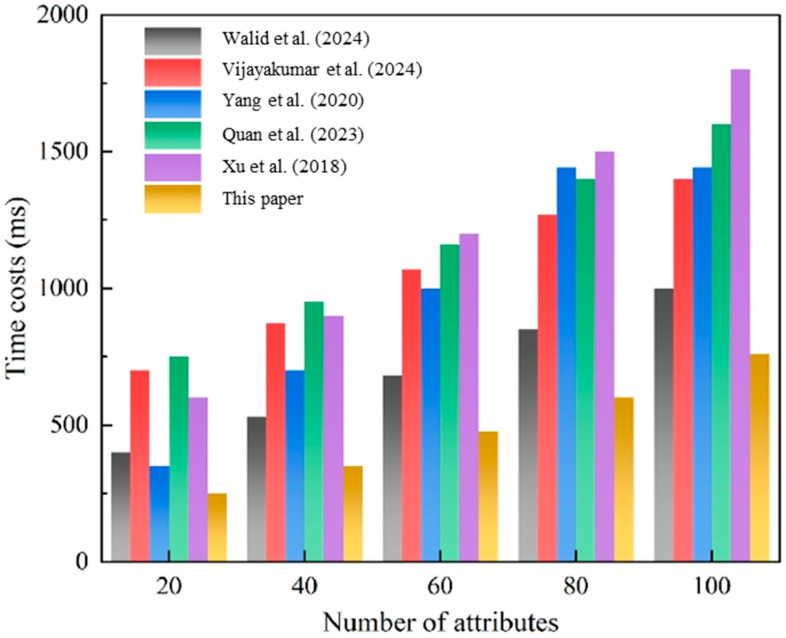
Designcryption after message dissemination. This figure compares the time overhead of the designcryption process after message dissemination. The performance evaluation is based on the work of Walid et al. [3], Vijayakumar et al. [24], Yang et al. [18], Quan et al. [13] and Xu et al. [21].

The designcryption performance comparison in Figure 6 reveals even more dramatic advantages. Our blockchain-assisted approach maintains less than 500 ms response times even at maximum attribute complexity, outperforming traditional schemes by 67–73%, with times of 1200–1500 ms. The distributed verification mechanism effectively eliminates the single-point computational bottlenecks present in references [13,21,24], while ensuring cryptographic integrity through our novel consensus protocol.

Overall, the numerical simulations show that our scheme outperforms the schemes in previous research in both the signcryption and designcryption phases. Specifically, in scenarios involving multi-attribute data, our scheme demonstrates greater scalability and computational efficiency, making it well-suited for IoT devices with limited resources.

## 5. Discussion

### 5.1. Security Model

The proposed framework employs a robust security model based on Attribute-Based Signcryption (ABSC) and blockchain technologies to secure medical data in an Internet of Medical Things (IoMT) environment. The ABSC mechanism ensures that only authorized entities, validated by specific attributes, can access the encrypted data. The security model incorporates the following key elements:

1. Data confidentiality: The ciphertext-policy ABSC ensures that only devices possessing the correct attribute sets and corresponding private keys can decrypt the data. This prevents unauthorized access to sensitive medical data.
2. Data integrity: The system uses cryptographic signatures ST to ensure that the data have not been tampered with during storage or transmission. Any modifications to the data can be detected through signature verification.
3. Message authenticity: The system guarantees that data access and transmission requests come from verified users. The proposed cryptographic mechanism is implemented through publicly accessible encryption keys issued by trusted authorities (TAs), which concurrently establish an authentication framework for both data requesters (DRs) and their associated smart devices (SDs).
4. Access control: The system provides fine-grained access control through an attribute set, ensuring that only authorized users can access the encrypted data. Each data access request is validated based on the user’s attributes, ensuring precise data retrieval permissions.

### 5.2. Threat Model

The Threat Model of this system identifies and mitigates several key security threats that could compromise the confidentiality, integrity, or availability of sensitive medical data. These include:

1. Identity forgery: An attacker could try to impersonate a legitimate user to gain access to the system. To prevent this, the system relies on the authentication provided by the trusted authorities (TAs), which ensure that only legitimate users receive private keys and are granted access to the system. The blockchain records every access request, which can be used for auditing and detecting any fraudulent activity.
2. Unauthorized access: Attackers may attempt to gain unauthorized access to medical data. However, this threat is mitigated by the ABSC mechanism, which enforces fine-grained access control based on user attributes. Only users who possess the correct attributes are allowed to decrypt and access the data. Additionally, the use of blockchain ensures that any unauthorized attempts to modify or access data will be easily detectable.
3. Data integrity attacks: Attackers may attempt to alter or tamper with data stored in the system. The use of blockchain ensures that any changes to the data are impossible without detection, as every change is recorded in an immutable ledger. In addition, the cryptographic signatures used in the ABSC scheme guarantee that the data remains unchanged during storage and transmission.
4. Availability attacks: The decentralized nature of the blockchain mitigates the risk of Denial-of-Service (DoS) or Distributed Denial-of-Service (DDoS) attacks, as there is no single point of failure in the system. Additionally, the edge computing infrastructure offloads data processing, reducing the burden on any single node and improving the system’s resilience against availability attacks.
5. Eavesdropping and data leakage: Even if an attacker intercepts communication, they cannot read the data without possessing the correct decryption keys. The encryption provided by ABSC ensures that the data are protected during transmission. Furthermore, the use of blockchain ensures the confidentiality of the encryption keys, making it difficult for attackers to obtain them.
6. Replay attacks: The system ensures protection against replay attacks by utilizing secure, time-stamped messages and access control protocols that verify each transaction’s authenticity before processing it.
7. Malicious insider attacks: Insiders, such as compromised edge devices or service providers, could attempt to access or tamper with the data. However, the use of blockchain ensures full transparency and auditability of all actions, enabling the detection of any malicious activities. Additionally, the ABSC mechanism ensures that only authorized users can access the data, and any unauthorized access will be flagged.

By integrating blockchain, edge computing, and ABSC, the proposed framework establishes a robust defense against common and advanced security threats in IoMT environments. These elements work together to ensure that patient data remains secure, private, and accessible only to authorized users, while offering transparency and traceability for audit and compliance purposes.

## 6. Conclusions

This paper proposes a novel framework integrating blockchain, edge computing, and Attribute-Based Signcryption (ABSC) to secure and efficiently manage Electronic Health Records (EHR) in the Internet of Medical Things (IoMT) environment. By leveraging the immutability of blockchain, the low latency of edge computing, and the fine-grained access control of ABSC, the system ensures strong data security and efficient management of EHRs.

However, several challenges remain. As medical data volumes increase, the computational and storage capabilities of edge computing could become a bottleneck. The rapid expansion of blockchain storage demands, driven by growing data access records, may strain network bandwidth and node processing capacity. Additionally, complex user attribute sets could lead to complicated ciphertext policies, resulting in increased computational costs, which may affect system performance, particularly in large-scale healthcare environments.

Future work will focus on optimizing the ABSC scheme and exploring lightweight encryption algorithms to reduce computational overhead. Strategies such as hierarchical or off-chain storage technologies will be examined to alleviate the storage pressure on blockchain. Moreover, multi-layered edge computing architectures will be explored to distribute computational tasks and improve system responsiveness. Finally, the integration of more flexible access control mechanisms, such as dynamic permission adjustment via machine learning, will be investigated to enhance system adaptability and performance.

## Figures and Tables

**Table 1:** System entities and their functions.

Component	Primary Role	Key Responsibilities
Smart device (SD)	Data collection	-Collects physiological data -Performs lightweight encryption
Edge device (ED)	Local processing/storage	-Processes and stores encrypted data -Maintains blockchain network
Service provider (SP)	Data analysis intermediary	-Processes medical data -Validates blockchain transactions
Trusted authority (TA)	Security management	-Manages authentication
Data owner (DO)	Data control	-Defines access policies -Records data transactions on blockchain
Data requester (DR)	Data access	-Requests data access -Undergoes identity verification

**Table 2:** Computational overhead analysis.

System Initialization	Registration	Signcryption	Designcryption	Reference
3$T_{e}$ + $T_{p}$	2|N|$T_{h}$ + 6$T_{e}$ + 2$T_{m}$	(5|N| + 6)$T_{e}$ + (2|N| + 4)$T_{m}$	2|N|$T_{e}$ + 3$T_{h}$ + |N|$T_{m}$	[31]
$3T_{e}+3T_{h}$	(3 + |N|)$T_{e}$ + |N|$T_{h}$	(3 + |N|)$T_{e}$ + (|N| + 1)$T_{h}$ + 2$T_{p}$	(|N| + 2)$T_{e}$ + (|N| + 1)$T_{h}$ + 2$T_{p}$	[24]
$T_{e}+T_{h}+T_{p}$	2|N|$T_{e}$ + 6$T_{h}$ + 2$T_{p}$	(5|N| + 6)$T_{e}$ + (2|N| + 4)$T_{h}$	2|N|$T_{e}$ + 3$T_{h}$ + |N|$T_{p}$	[3]
3$T_{e}$ + $T_{p}$	(3 + |N|)$T_{e}$ + |N|$T_{m}$	(2|N| + 6)$T_{e}$ + (|N| + 1)$T_{m}$ + 2$T_{h}$	(|N|+2)$T_{e}$ + (|N| + 1)$T_{m}$ + 2$T_{h}$	This paper

## Data Availability

Data are contained within the article.

## Abbreviations

The following abbreviations are used in this manuscript:

SD	Smart devices
ED	Edge devices
TA	Trusted authorities
SP	Services providers
DO	Data owners
DR	Data eequesters
ABSC	Attribute-Based Signcryption
IoMT	Internet of Medical Things
CP-ABSC	ciphertext-policy Attribute-Based Signcryption
ABE	Attribute-Based Encryption
IoT	Internet of Things
EHR	Electronic Health Record
ECC	Elliptic Curve Cryptography
